# Ogilvie Syndrome Revealing a Pheochromocytoma: A Rare Intersection of Two Entities

**DOI:** 10.7759/cureus.102171

**Published:** 2026-01-23

**Authors:** Zineb Boukhal, Malak Afifi, Yasmin Tahiri, Fatima Zahra El Rhaoussi, Mohamed Tahiri, Fouad Haddad, Wafaa Hliwa, Ahmed Bellabah, Badre Wafaa, Kenza Berrada, Chorouk Mountassir, Ghizlane Lembarki, Samira Lazer

**Affiliations:** 1 Gastroenterology and Hepatology, Ibn Rochd University Hospital Center, Casablanca, MAR; 2 Faculty of Medicine and Pharmacy, Hassan II University, Casablanca, MAR; 3 Central Radiology Department, Ibn Rochd University Hospital Center, Casablanca, MAR

**Keywords:** catecholamines, cecostomy, endoscopic colonic decompression, hypertensive crisis, ogilvie syndrome, pheochromocytoma

## Abstract

Pheochromocytoma is a rare neuroendocrine tumor arising from the chromaffin cells of the adrenal medulla and characterized by excessive catecholamine secretion. Gastrointestinal manifestations are uncommon and may occasionally lead to the diagnosis. We report the case of a 33-year-old woman presenting with acute intestinal obstruction secondary to Ogilvie syndrome, which ultimately revealed an underlying pheochromocytoma. Imaging demonstrated a left adrenal mass, and biochemical assays confirmed elevated catecholamine levels. Despite appropriate initial management, the patient developed a fatal hypertensive crisis. This case highlights the importance of recognizing pseudo-obstructive syndromes as rare but life-threatening presentations of pheochromocytoma.

## Introduction

Pheochromocytoma is a rare neuroendocrine tumor originating from the chromaffin cells of the adrenal medulla and characterized by excessive catecholamine secretion. Its estimated annual incidence ranges from two to eight cases per million inhabitants. The disease most often affects young adults and is commonly diagnosed during the evaluation of sustained or paroxysmal hypertension. Clinically, it is classically associated with Ménard’s triad - episodic headache, sweating, and palpitations - which reflects catecholamine excess. Surgical excision is the only curative treatment and requires careful preoperative medical preparation to minimize perioperative cardiovascular complications [1,2].

Digestive manifestations of pheochromocytoma are rare and frequently underrecognized. When present, they may include nonspecific symptoms such as abdominal pain, constipation, or ileus, and only exceptionally present as acute intestinal pseudo-obstruction. Ogilvie syndrome, also known as acute colonic pseudo-obstruction, is defined by marked colonic dilatation in the absence of any mechanical obstruction. It is typically encountered in hospitalized or critically ill patients and is thought to result from autonomic dysfunction affecting colonic motility [3].

The coexistence of pheochromocytoma and Ogilvie syndrome is exceptionally uncommon and has been reported only sporadically in the literature. Excessive catecholamine secretion may profoundly inhibit intestinal peristalsis through α-adrenergic stimulation, leading to acute colonic pseudo-obstruction [4,5]. We report a case in which Ogilvie syndrome was the revealing manifestation of an underlying pheochromocytoma, highlighting a rare but potentially life-threatening clinical scenario.

## Case presentation

We present the case of a 33-year-old woman with a two-year history of hypertension treated with amlodipine. She was initially admitted to the emergency department with symptoms consistent with intestinal obstruction.

The patient reported a one-year history of chronic constipation that had worsened three days before admission, manifesting as complete cessation of bowel movements and flatus, accompanied by abdominal pain and vomiting. In addition, she reported episodic pulsatile headaches, profuse sweating, and palpitations that had previously gone unrecognized during hypertension management and had become more frequent and pronounced over the two months preceding admission, constituting the classic Ménard triad.

On clinical examination, the patient was conscious and hemodynamically stable, though markedly hypertensive at 200/120 mmHg, with a tachycardic pulse of 112 beats per minute. Her respiratory status was normal. Abdominal examination revealed marked distension with diffuse tympany on percussion, but no signs of peritoneal irritation were observed. Laboratory investigations showed moderate hyponatremia (serum sodium 128 mmol/L), which was promptly corrected with appropriate medical management; no potassium imbalance was detected (Table 1).

**Table 1:** Laboratory findings

Test name (unit)	Observed value	Normal range
Serum sodium (mmol/L)	128	135–145
Serum potassium (mmol/L)	Normal	3.5–5.0
24-hour urinary metanephrines (µg/24 h)	278	40–150
24-hour urinary normetanephrines (µg/24 h)	1200	110–320

**Figure 1:**
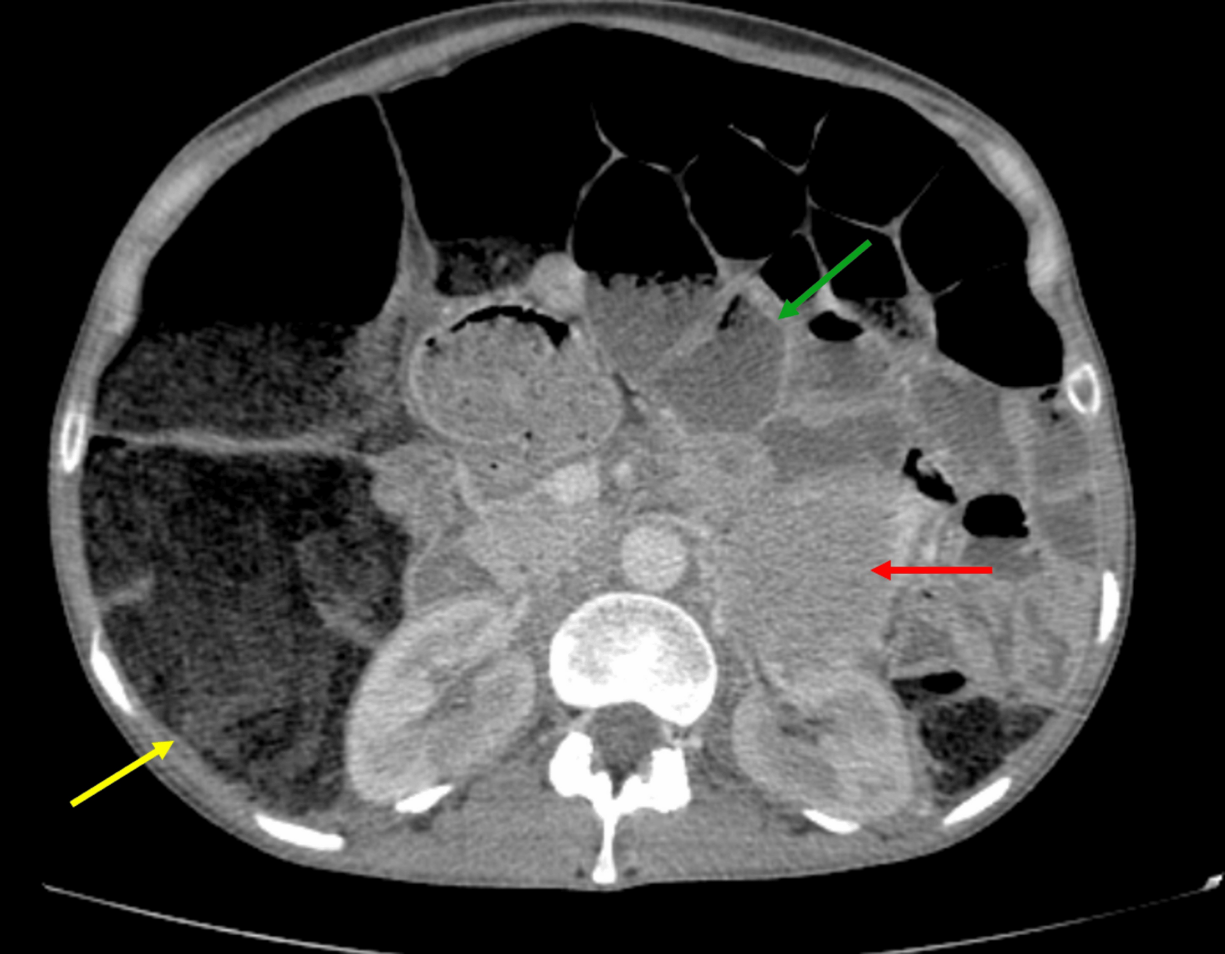
Contrast-enhanced axial abdominal CT showing marked colonic (yellow arrow) and small-bowel distension (green arrow), with a heterogeneous left adrenal mass (red arrow)

**Figure 2:**
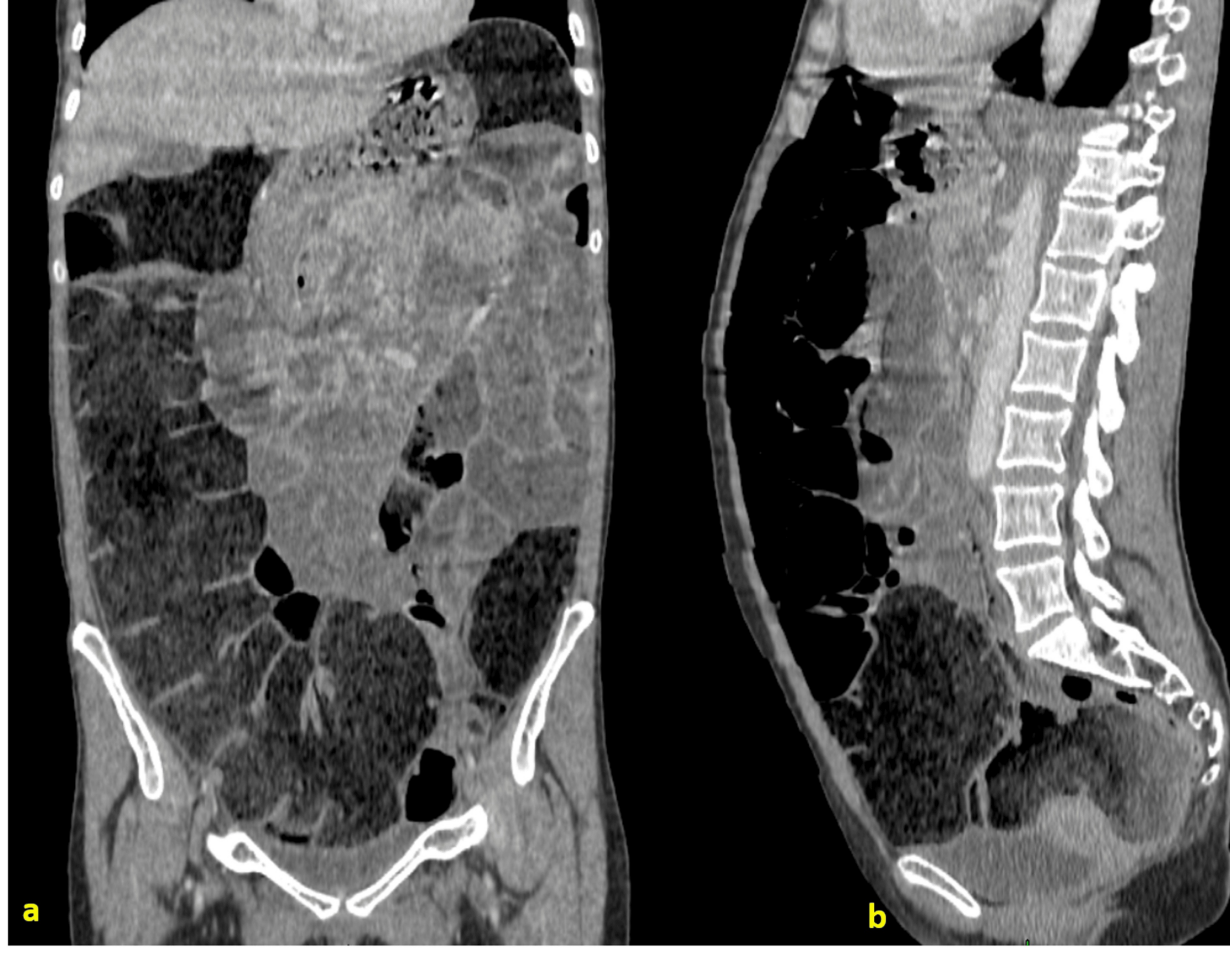
Contrast-enhanced abdominal CT, coronal and axial views, showing diffuse colonic and small-bowel distension without any identifiable transition point a: Coronal view b: Axial view

An abdominal X-ray revealed diffuse colonic distension. A subsequent CT scan confirmed extensive colonic dilation, with the cecum measuring 80 mm in diameter, and no identifiable transition zone. Additionally, a left retroperitoneal mass was detected, raising suspicion for Ogilvie syndrome potentially associated with a pancreatic tumor or adrenal mass (Figures 1, 2).

**Figure 3:**
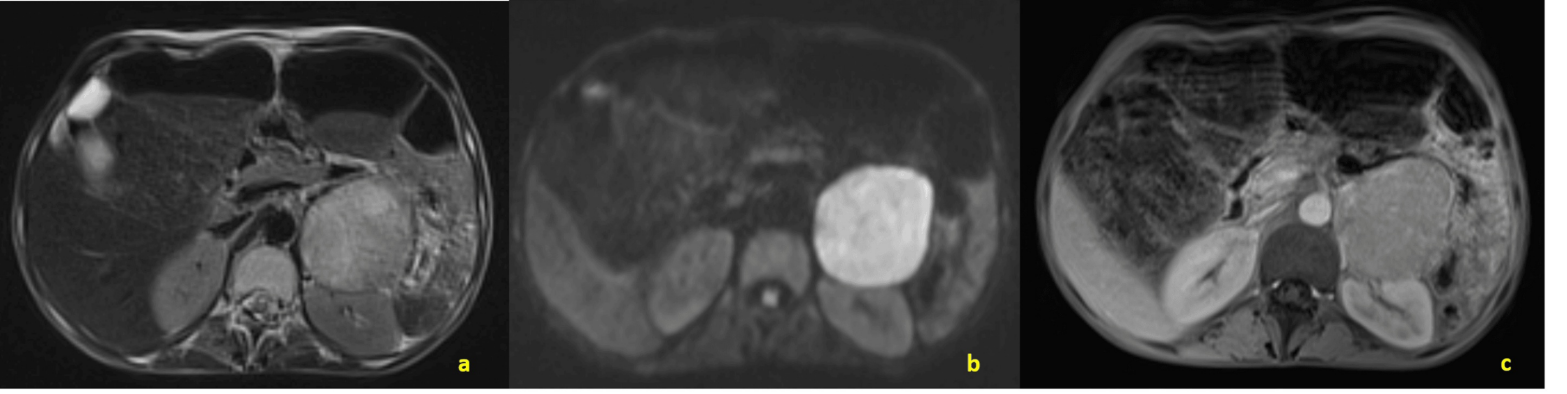
Abdominal MRI (axial views) of the left adrenal mass, including T2-weighted, diffusion-weighted, and post-gadolinium T1 sequences, showing heterogeneous hyperintensity on T2 and diffusion images and heterogeneous enhancement after gadolinium administration a: Axial T2-weighted image showing a well-circumscribed left adrenal mass with heterogeneous hyperintensity b: Diffusion-weighted imaging demonstrating restricted diffusion within the lesion c: Post-gadolinium T1-weighted image revealing heterogeneous enhancement of the adrenal mass, consistent with pheochromocytoma

**Figure 4:**
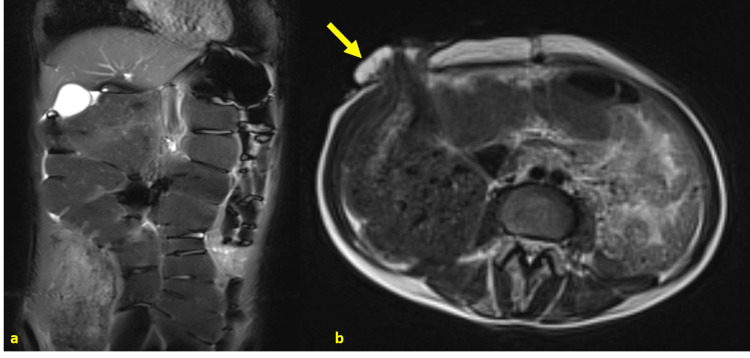
T2-weighted MRI (coronal and axial views) showing diffuse colonic distension. Note the right colostomy opening a: coronal view b: axial view Yellow arrow: right colostomy opening

Further investigation with abdominal MRI identified a well-circumscribed, rounded mass in the left adrenal region. The mass appeared isointense on T1-weighted images, heterogeneously hyperintense on T2-weighted and fat-suppressed sequences, and showed heterogeneous enhancement after gadolinium administration. The lesion measured 62 mm in diameter and was highly suggestive of pheochromocytoma (Figures 3, 4).

Laboratory analysis revealed significantly elevated 24-hour urinary catecholamines, with metanephrines at 278 μg (normal: 40-150 μg) and normetanephrines at 1200 μg (normal: 110-320 μg) (Table 1).

Initial management of Ogilvie syndrome included two attempts at endoscopic colonic decompression, both unsuccessful due to extrinsic compression at the left colonic flexure by the adrenal mass. The patient subsequently underwent surgical cecostomy, which provided temporary symptom relief.

Approximately two weeks after the cecostomy, while awaiting medical optimization prior to definitive surgical resection of the pheochromocytoma, the patient developed an uncontrolled hypertensive crisis that rapidly led to hemodynamic deterioration and ultimately resulted in death, despite appropriate medical management.

## Discussion

Pheochromocytomas are rare catecholamine-secreting tumors originating from chromaffin cells of the adrenal medulla. They release adrenaline, noradrenaline, and dopamine, which exert their physiological effects through stimulation of adrenergic (α and β) and dopaminergic (DA₁ and DA₂) receptors [2]. Excess circulating catecholamines account for the classical triad of paroxysmal headache, sweating, and palpitations described by Ménard, a triad that was fully present in our patient [1,2]. In this case, it is likely that the surgical stress related to the cecostomy, performed before adequate alpha-adrenergic blockade, precipitated a state of heightened catecholamine release, thereby predisposing the patient to the subsequent fatal hypertensive crisis.

Ogilvie syndrome, or acute colonic pseudo-obstruction (ACPO), is characterized by marked colonic dilatation without any mechanical obstruction or inflammatory cause. This excludes mechanical dilations caused by upstream obstructions or those associated with acute colitis, ischemic colitis, or cryptogenic conditions such as toxic megacolon or reflex ileus accompanying peritonitis [3].

Its exact pathophysiology remains incompletely understood and is thought to involve autonomic imbalance between sympathetic inhibition and parasympathetic stimulation of colonic motility [3]. Multiple contributing factors exist including neurological, vascular, hormonal, and pharmacological mechanisms. In the neurogenic theory, colonic motor activity is regulated by the interaction between the sympathetic nervous system, which has an inhibitory function, and the parasympathetic nervous system, which plays an excitatory role [3].

The association between pheochromocytoma and Ogilvie syndrome is exceptional. Several case reports and reviews have described intestinal pseudo-obstruction as an uncommon manifestation of catecholamine-secreting tumors [4,5]. The proposed mechanism involves excessive catecholamine release, which suppresses intestinal peristalsis and smooth-muscle contractility through α-adrenergic stimulation, thereby reducing intestinal tone and motility [6]. This neurogenic inhibition may be further exacerbated by vascular spasm or altered splanchnic blood flow [7,8].

In our case, the coexistence of a functional pheochromocytoma with a complete Ménard triad, severe hypertension, and the absence of any mechanical or inflammatory etiology is consistent with a catecholamine-mediated autonomic dysfunction contributing to the development of acute colonic pseudo-obstruction.

Clinically, Ogilvie syndrome typically presents with progressive abdominal distension, hyper-resonance on percussion, nausea, vomiting, abdominal pain, and cessation of bowel movements, closely mimicking distal mechanical intestinal obstruction [2]. The occurrence of fever or increasing abdominal tenderness should raise concern for complications such as colonic ischemia or perforation.

Although laboratory investigations have limited diagnostic value in isolation, imaging plays a central role in establishing the diagnosis and excluding differential causes. Plain abdominal radiography is usually the first-line examination, characteristically demonstrating diffuse colonic dilatation extending from the cecum to the rectosigmoid junction. Cecal diameter is a key prognostic parameter, as increasing distension is strongly associated with the risk of perforation. Contrast-enhanced abdominal computed tomography is essential to exclude alternative diagnoses, including mechanical obstruction, sigmoid or cecal volvulus, fecal impaction, neoplasia, or peritonitis. The presence of pneumoperitoneum, peritoneal effusion, or colonic pneumatosis on CT imaging strongly suggests perforation and warrants urgent surgical evaluation [9,10].

In our patient, computed tomography confirmed massive colonic dilatation consistent with Ogilvie syndrome and simultaneously revealed a retroperitoneal mass suggestive of adrenal origin. Further characterization by MRI demonstrated radiological features typical of pheochromocytoma, while markedly elevated urinary catecholamines provided biochemical confirmation. Taken together with the clinical presentation - marked by severe hypertension and a complete Ménard triad - these findings support a unifying pathophysiological mechanism, whereby catecholamine excess induces autonomic dysregulation and inhibition of colonic motility, thereby contributing to the development of acute colonic pseudo-obstruction [4,11].

There is no universal consensus regarding the maximum tolerable cecal diameter. However, perforation risk increases significantly when the cecum exceeds 9 cm, and up to 25% of patients perforate beyond 12 cm [9,12]. Management should therefore be guided by clinical severity and colonic distension.

In the absence of peritoneal signs, conservative management is recommended, including bowel rest, nasogastric and rectal decompression, correction of electrolytes, and discontinuation of motility-suppressing drugs [10]. Pharmacological treatment with intravenous neostigmine may be effective in refractory cases. Endoscopic decompression is considered when conservative therapy fails, but recurrence rates remain significant [13].

In our patient, repeated endoscopic decompression attempts were unsuccessful because of extrinsic compression of the left colonic flexure by the adrenal mass, leading to surgical cecostomy and only transient clinical improvement [4,5,7,8]. According to the literature, complete surgical excision is the definitive treatment for pheochromocytoma and must be preceded by appropriate preoperative medical preparation, primarily based on α-adrenergic blockade, to reduce the risk of catecholamine-induced cardiovascular complications. This case illustrates the persistent risk of severe, potentially fatal hypertensive events in patients with marked catecholamine excess, particularly when diagnosis and definitive treatment are delayed, as reported in similar cases from the literature [14].

## Conclusions

This case illustrates a rare presentation of pheochromocytoma revealed by acute colonic pseudo-obstruction. Excess catecholamine secretion likely contributed to severe autonomic dysfunction, leading to refractory intestinal dilation and subsequent cardiovascular instability. Despite decompressive interventions, the delayed recognition of the underlying endocrine cause preceded a fatal hypertensive crisis. This report highlights that in any patient presenting with acute colonic pseudo-obstruction, particularly in the presence of hypertension or adrenergic symptoms, pheochromocytoma should be actively excluded through appropriate biochemical testing before surgical intervention, when clinically feasible, as early diagnosis may help prevent fatal outcomes.
